# Association of Early Hysterectomy With Risk of Cardiovascular Disease in Korean Women

**DOI:** 10.1001/jamanetworkopen.2023.17145

**Published:** 2023-06-12

**Authors:** Jin-Sung Yuk, Byung Gyu Kim, Byoung Kwon Lee, Jongkwon Seo, Gwang Sil Kim, Kyongjin Min, Hye Young Lee, Young Sup Byun, Byung Ok Kim, Seung-Woo Yang, Myoung-Hwan Kim, Sang-Hee Yoon, Yong-Soo Seo

**Affiliations:** 1 Department of Obstetrics and Gynecology, Sanggye Paik Hospital, Inje University College of Medicine, Seoul, Republic of Korea; 2 Division of Cardiology, Department of Internal Medicine, Sanggye Paik Hospital, Inje University College of Medicine, Seoul, Republic of Korea; 3 Division of Cardiology, Department of Internal Medicine, Gangnam Severance Hospital, Yonsei University College of Medicine, Seoul, Korea

## Abstract

**Importance:**

Women who undergo surgical hysterectomy before natural menopause may have an earlier increase in hematocrit and storage iron levels than those who continue menstruation, thereby increasing the risk of cardiovascular disease (CVD) at ages younger than usually seen. Examining this issue may provide important implications for women’s cardiovascular health to both physicians and patients.

**Objective:**

To evaluate the association of hysterectomy with the risk of incident CVD among women before age 50 years.

**Design, Setting, and Participants:**

In this Korean population-based cohort study, 135 575 women aged 40 to 49 years were evaluated from January 1, 2011, to December 31, 2014. After propensity score matching in covariates including age, socioeconomic status, region, Charlson Comorbidity Index, hypertension, diabetes, dyslipidemia, menopause, menopausal hormone therapy, and adnexal surgery before inclusion, 55 539 pairs were included in the hysterectomy and nonhysterectomy groups. Participants were followed up until December 31, 2020. Data analysis was conducted from December 20, 2021, to February 17, 2022.

**Main Outcomes and Measures:**

The primary outcome was an incidental CVD, a composite of myocardial infarction, coronary artery revascularization, and stroke. The individual components of the primary outcome were also evaluated.

**Results:**

A total of 55 539 pairs were included; median age in the combined groups was 45 (IQR, 42-47) years. During median follow-up periods in the hysterectomy group of 7.9 (IQR, 6.8-8.9) years and nonhysterectomy group of 7.9 (IQR, 6.8-8.8) years, the incidence of CVD was 115 per 100 000 person-years for the hysterectomy group and 96 per 100 000 person-years for the nonhysterectomy group. After adjusting for confounding factors, the hysterectomy group had an increased risk of CVD compared with the nonhysterectomy group (hazard ratio [HR], 1.25; 95% CI, 1.09-1.44). The incidences of myocardial infarction and coronary artery revascularization were comparable between the groups, whereas the risk of stroke was significantly higher in the hysterectomy group (HR, 1.31; 95% CI, 1.12-1.53). Even after excluding women who underwent oophorectomy, the hysterectomy group had higher risks of CVD (HR, 1.24; 95% CI, 1.06-1.44).

**Conclusions and Relevance:**

The findings of this cohort study suggest early menopause owing to hysterectomy was associated with increased risks for a composite of CVD, particularly stroke.

## Key Points

**Question:**

Is early hysterectomy before natural menopause in women associated with an increased risk of cardiovascular disease (CVD)?

**Findings:**

In this Korean nationwide cohort study including 135 575 women, early surgical hysterectomy before age 50 years was independently associated with an increased risk of CVD, especially stroke. Even after excluding women who underwent oophorectomy, the hysterectomy group had higher risks of CVD than the nonhysterectomy group.

**Meaning:**

This study noted an association between early hysterectomy and an increased risk of CVD; because the incidence of CVD was not high, a change in clinical practice may not be needed.

## Teaser

This cohort study examines the incidence of cardiovascular disease in Korean women who undergo hysterectomy when younger than 50 years.

## Introduction

Cardiovascular disease (CVD) is the leading cause of morbidity and mortality in women and affects up to 36% of all women worldwide.^1,2^ The risk of CVD rapidly increases after menopause, and early menopause is associated with an increased risk of coronary artery disease and stroke.^3,4,5^ The increasing incidence of CVD in postmenopausal women may be due to lower female sex hormone levels, which have cardiovascular protective effects.^6^ However, hemorheologic changes that occur after menopause may also affect the occurrence of CVD. Women’s hematocrit levels increase substantially as women enter into the fifth decade of life, which is the average age of menopause.^7^ Elevated hematocrit levels increase whole blood viscosity, which can lead to endothelial injury, rupture of plaques by increasing shear stress on the vessel wall, and thrombus formation by red cell aggregation.^8,9,10^ An increase in hematocrit levels is also associated with an increase in iron and ferritin levels, which are prooxidant cofactors linked to the production of hydroxy radicals and progression of atherosclerosis.^11^ Women who undergo hysterectomy before natural menopause may have an earlier increase in hematocrit and storage iron levels than those who continue menstruation, thereby increasing the risk of early CVD.^12^

Examining the use of hysterectomy at a younger age and women’s risk of CVD is important since it is a commonly performed gynecologic procedure with well-documented benefits in relieving symptoms and improving quality of life.^13^ Thus, this nationwide, population-based cohort study conducted in Korea to evaluate the risk of incident CVD between women with and without hysterectomy before age 50 years after propensity score matching.

## Methods

Because the Korean National Health Insurance Service (NHIS) is a universal health coverage system in South Korea and is necessarily linked to all Korean medical facilities, the NHIS database contains medical information (age, sex, diagnosis code, prescription drug information, surgical procedure code, type of medical insurance, hospitalization, and outpatient records) of the entire Korean population (approximately 51 million individuals).^14^ The Korean Health Insurance Review and Assessment Service (HIRA) is an institution that screens whether medical expenses claimed by medical institutions are clinically valid and shares a significant part of NHIS data. The HIRA data are publicly available and can be requested from the HIRA data site.^15^ This population-based, retrospective cohort study comprised health insurance data provided by the HIRA from January 1, 2007, to December 31, 2020. The study protocol conformed with the ethical guidelines of the 1975 Declaration of Helsinki^16^and was approved by the institutional review board of Sanggye Paik Hospital. All personal information was deidentified, and the analysis was possible only using a virtual server within the HIRA. Therefore, the requirement for patient informed consent was waived according to the Bioethics and Safety Act of South Korea. This study followed the Strengthening the Reporting of Observational Studies in Epidemiology (STROBE) reporting guideline.

### Selection of Participants

**Figure 1.**
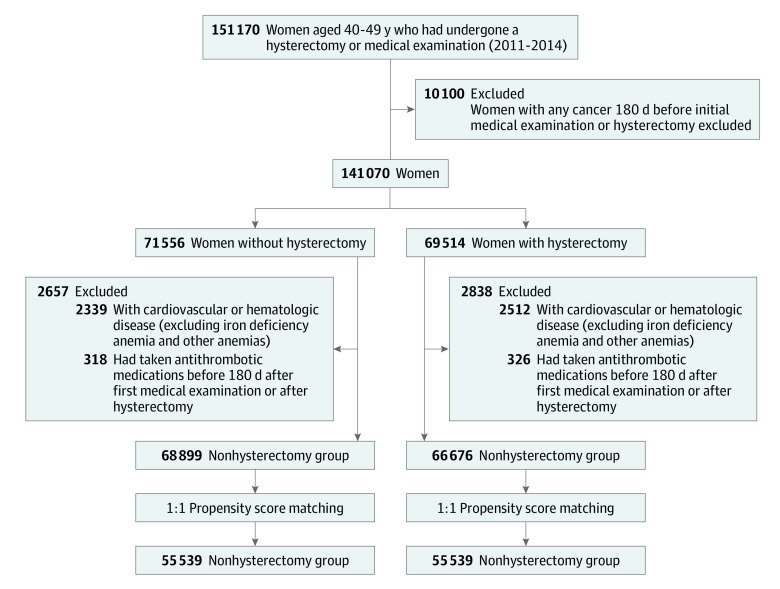
Selection of Participants From the Korean Health Insurance Review and Assessment Service Data Age per 5-year interval, year at inclusion, socioeconomic status, region, Charlson Comorbidity Index, hypertension, diabetes, dyslipidemia, menopause before inclusion, menopausal hormone therapy before inclusion, and adnexal surgery before inclusion were used in propensity matching.

We used diagnostic codes included in the claims that were classified as per the *International Statistical Classification of Diseases and Related Health Problems, 10th Revision* (*ICD-10*), the surgical and treatment codes from the Health Insurance Medical Care Expenses (2016 and 2019 version) for selection of participants, extraction of variables, and assessment of outcomes. Participant flow and reasons for exclusion are provided in Figure 1. In this study, for the case and control groups, women aged 40 to 50 years in 2011 to 2014 were screened. The hysterectomy group included patients who underwent hysterectomy owing to uterine myoma or adenomyosis from January 1, 2011, to December 31, 2014. The date of hysterectomy was considered the inclusion day for these patients. The control group included women who visited a medical institute for health check-ups during the period. Among this population, patients with a history of hysterectomy after 2014 were excluded. The first visit date to a medical institute for health examination was considered the inclusion day of the control group. Patients with an *ICD-10* code of cancer (any Cxx codes), CVD (codes I21-23, I60-64), and hematologic disease (codes D50-89) except for iron deficiency anemia (code D50), anemia after acute bleeding (code D62), anemia with chronic disease (code D63), and other anemia (code D64) before 180 days of inclusion were excluded from both groups. In the selected hysterectomy and control groups, 1:1 propensity matching was conducted for age at 5-year intervals, year at inclusion, socioeconomic status, region, Charlson Comorbidity Index (CCI) score, hypertension, diabetes, dyslipidemia, menopause before inclusion, menopausal hormone therapy (MHT) before inclusion, and adnexal surgery before inclusion. The accuracy of coding of menopause has not been well validated; however, a previous study reported that it is not overestimated compared with other studies.^17^ The CCI assigns constant weights from 1 to 6 for 19 diseases defined through medical record investigations, and then corrects the sum of these weights.^18^ Women were followed up from the inclusion day to death from any cause or to the end of the study (December 31, 2020).

### Cardiovascular Outcomes

The primary outcome was incidental CVD, which was defined as the first hospitalization or death for myocardial infarction (MI), coronary artery revascularization, or stroke. The individual components of the primary outcome were also evaluated. Myocardial infarction was defined as records of *ICD-10* codes I21 to 23 during an inpatient visit. Coronary artery revascularization was defined as a record of receiving percutaneous coronary intervention (transluminal coronary angioplasty, transcatheter placement of intracoronary stent, transluminal coronary atherectomy, transluminal angioplasty, intravascular installation of metallic stent, and thrombus removal) during an inpatient visit. To identify the participants’ incident stroke events, we investigated cerebral bleeding and infarction (*ICD-10* code I60–64) after excluding transient ischemic attacks (code G45) or other kinds of thromboembolisms. We only considered hospitalization or deaths with the above-listed diagnoses as primary diagnoses of the hospitalization or the primary causes of death.

### Variables

The receipt of medical aid as medical insurance served as a proxy for socioeconomic status. The region was classified as an urban area if the administrative district was metropolitan city level or larger and a rural area if the administrative district was nonmetropolitan. The CCI score was obtained using diagnostic codes from the date of inclusion to the previous 1 year.^19^ Hypertension (code I10-15), diabetes (code E10-14), dyslipidemia (code E78), and menopause (codes N95, N80.0, M81.0, and E28.3) were defined as visiting medical institutes more than 2 times with the corresponding codes before the study inclusion day. A history of adnexal surgery was defined if any surgery of extirpation of adnexal tumor (unilateral or bilateral salpingoophorectomy, unilateral or bilateral oophorectomy, unilateral or bilateral salpingectomy, and unilateral or bilateral ovarian cystectomy), ovarian wedge resection, incision and drainage of ovarian cyst, or adnexectomy for adhesion was performed more than once. Receiving MHT (tibolone, estradiol valerate, estradiol hemihydrate, dydrogesterone, norethisterone acetate, medroxyprogesterone acetate, drospirenone, or cyproterone) more than 180 days before inclusion was considered as MHT before inclusion. When the first MHT was used after inclusion, it was considered as MHT after inclusion.

### Statistical Analysis

Data analysis was conducted from December 20, 2021, to February 17, 2022. Continuous variables are reported as medians (IQRs), and categorical variables are expressed as numbers and frequencies. Continuous variables were analyzed using the Wilcoxon signed rank test, and categorical variables were compared using the Cochran-Mantel-Haenszel test. Standardized difference was used to evaluate matching variables. An absolute standardized difference less than 0.1 for the matching variables indicated an appropriate balance between the groups.^20^ Stratified Cox proportional hazards regression analysis was performed to analyze the association between hysterectomy and the clinical end points by confounding factors. Time-to-event data according to hysterectomy status were presented using Kaplan-Meier curves. For proportional hazards assumptions, the Schoenfeld residual test was used. There were no missing data regarding the baseline characteristics of the cohort that we can select since HIRA has national data linked to the resident registration number given by the state to all citizens. For the sensitivity test, a stratified Cox regression analysis was performed to compare the risk of CVD between women who underwent only laparoscopic hysterectomy in the hysterectomy group and the nonhysterectomy group. All tests were 2-sided, and the results were considered statistically significant at *P* < .05. These analyses were conducted using SAS Enterprise Guide, version 6.1 (SAS Institute Inc) and R Statistical software, version 3.5.3 (R Foundation for Statistical Computing).

## Results

### Baseline Characteristics

Overall, 135 575 women were included in this analysis (66 676 in the hysterectomy group and 68 899 in the nonhysterectomy group) (Figure 1). The baseline characteristics of the study population after propensity score matching are reported in Table 1. After propensity score matching, among the 55 539 matched pairs, the absolute standardized value between the hysterectomy and nonhysterectomy groups was less than 0.1. The median age of the matched hysterectomy and nonhysterectomy groups was 45 (IQR, 42-47) years, and 52 550 women (47.3%) were younger than 45 years. In the cohort, 8.3% of women had menopause before inclusion, and 1.1% of women were receiving MHT. A total of 1.7% of women underwent adnexal surgery before inclusion.

**Table 1.** Baseline Characteristics of Participants Based on Whether Hysterectomy Was Performed

Characteristic	Participants, No. (%)			Standardized difference
	Total (N = 111 078)	Nonhysterectomy (n = 55 539)	Hysterectomy (n = 55 539)	
Age, median (IQR), y	45 (42-47)	45 (42-47)	45 (43-47)	.09
Age at inclusion, y				
40-44	52 550 (47.3)	26 615 (47.9)	25 935 (46.7)	.03
45-49	58 528 (52.7)	28 924 (52.1)	29 604 (53.3)	
Year at inclusion				
2011	26 332 (23.7)	13 559 (24.4)	12 773 (23.0)	.05
2012	27 397 (24.7)	13 291 (23.9)	14 106 (25.4)	
2013	28 952 (26.1)	14 819 (26.7)	14 133 (25.4)	
2014	28 397 (25.6)	13 870 (25.0)	14 527 (26.2)	
Socioeconomic status				
Receipt of medical aid as medical insurance	3420 (3.1)	1758 (3.2)	1662 (3.0)	.01
Region				
Urban area	62 555 (56.3)	30 995 (55.8)	31 560 (56.8)	.02
Rural area	48 523 (43.7)	24 544 (44.2)	23 979 (43.2)	
CCI score				
0	85 071 (76.6)	42 501 (76.5)	42 570 (76.6)	.02
1	15 582 (14)	7960 (14.3)	7622 (13.7)	
≥2	10 425 (9.4)	5078 (9.1)	5347 (9.6)	
Hypertension	12 302 (11.1)	6065 (10.9)	6237 (11.2)	.01
Diabetes	7503 (6.8)	3756 (6.8)	3747 (6.7)	<.001
Dyslipidemia	18 490 (16.6)	9268 (16.7)	9222 (16.6)	.002
Menopause before inclusion	9215 (8.3)	4672 (8.4)	4543 (8.2)	.008
MHT before inclusion	1187 (1.1)	569 (1)	618 (1.1)	.009
Adnexal surgery before inclusion	1899 (1.7)	952 (1.7)	947 (1.7)	<.001
First MHT after inclusion	11 146 (10)	3856 (6.9)	7290 (13.1)	<.001

Abbreviations: CCI, Charlson Comorbidity Index; MHT, menopausal hormone therapy.

### Cardiovascular Outcomes

**Figure 2.**
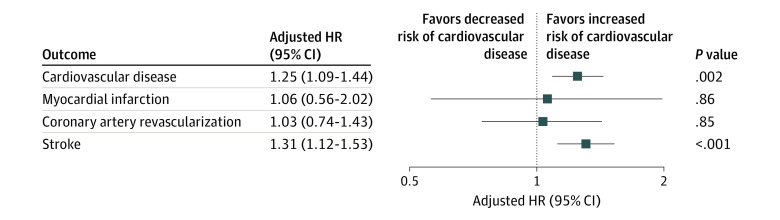
Hazard Ratios (HRs) for Risk of Adverse Cardiovascular Events Adjustments made for age, socioeconomic status, region, Charlson Comorbidity Index, hypertension, diabetes, dyslipidemia, menopause before inclusion, menopausal hormone therapy before inclusion, adnexal surgery before inclusion, and menopausal hormone therapy after inclusion.

The incidence and risk of cardiovascular outcomes between the groups after propensity score matching are presented in Table 2 and eTable 1 in Supplement 1. During the follow-up period (hysterectomy group: median, 7.9 [IQR, 6.8-8.9] years; nonhysterectomy group: median, 7.9 [IQR, 6.8-8.8] years), the incidence of CVD was 115 per 100 000 person-years for the hysterectomy group and 96 per 100 000 person-years for the nonhysterectomy group (eTable 1 in Supplement 1). The hysterectomy group had an increased risk of CVD compared with the nonhysterectomy group (hazard ratio [HR], 1.25; 95% CI, 1.09-1.44; *P* = .002) (Table 2 and Figure 2). For individual outcomes, the incidence of MI and coronary revascularization were comparable between the groups, whereas the risk of stroke was significantly higher in the hysterectomy group than in the nonhysterectomy group (HR, 1.31; 95% CI, 1.12-1.53; *P* < .001).

**Table 2.** Incidence and Risk of Cardiovascular Outcomes in the Hysterectomy and Nonhysterectomy Groups

Cardiovascular outcome	No. (%) of events		Unadjusted			Adjusted^a^		
	Nonhysterectomy (n = 55 539)	Hysterectomy (n = 55 539)	HR (95% CI)	*P* value	HR (95% CI)		*P* value
Cardiovascular disease	422 (0.8)	507 (0.9)	1.17 (1.03-1.34)	.02	1.25 (1.09-1.44)		.002
Myocardial infarction	26 (0)	25 (0)	0.96 (0.54-1.70)	.88	1.06 (0.56-2.02)		.86
Coronary artery revascularization	110 (0.2)	112 (0.2)	0.97 (0.74-1.27)	.84	1.03 (0.74-1.43)		.85
Stroke	322 (0.6)	406 (0.7)	1.24 (1.07-1.44)	.004	1.31 (1.12-1.53)		<.001

Abbreviation: HR, hazard ratio.

^a^ Adjusted for age, socioeconomic status, region, Charlson Comorbidity Index, hypertension, diabetes, dyslipidemia, menopause before inclusion, menopausal hormone therapy before inclusion, adnexal surgery before inclusion, and menopausal hormone therapy after inclusion.

### Sensitivity Analysis

**Figure 3.**
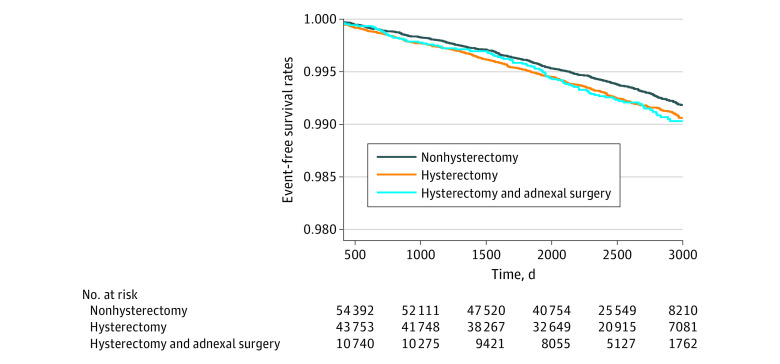
Cardiovascular Disease Events Over Time

To minimize the possible effect of female sex hormones, subgroup analysis was performed based on whether participants underwent adnexal surgery simultaneously with hysterectomy. Regardless of adnexal surgery, the results tended to be similar. Women who underwent hysterectomy without adnexal surgery had a higher risk of CVD than those who did not undergo hysterectomy (HR, 1.24; 95% CI, 1.06-1.44; *P* = .008) (eTable 2 in Supplement 1). This result was noted mainly with stroke (HR, 1.28; 95% CI, 1.08-1.52; *P* = .005). Kaplan-Meier curves showed that the CVD events gradually started to differ between hysterectomy and nonhysterectomy groups over time (Figure 3). Kaplan-Meier curves regarding MI or stroke are shown in the eFigure in Supplement 1. In addition, the outcomes of laparoscopic hysterectomy were evaluated. The characteristics of the women who underwent laparoscopic hysterectomy are presented in eTable 3 in Supplement 1. The risk of stroke in the laparoscopic hysterectomy group was significantly higher than that in the nonhysterectomy group (HR, 1.32; 95% CI 1.01-1.72; *P* = .04) (eTable 4 in Supplement 1). However, the difference of MI or coronary revascularization was not detected (eTable 4 in Supplement 1).

## Discussion

The present analysis from a nationwide, population-based, cohort study noted that women who underwent hysterectomy when younger than 50 years had an increased risk of incidental CVD, noted mainly with stroke, compared with those without hysterectomy. This risk increase was consistent even after adjusting for confounders and after excluding patients who underwent adnexal surgery simultaneously with hysterectomy.

Previous studies have provided evidence of the association between hysterectomy and CVD; however, the results are contradictory.^21,22,23,24,25^ Although a Swedish population study reported the association of hysterectomy in women younger than 50 years with an elevated risk of CVD, the result was limited because traditional cardiovascular risk factors were not adjusted in the Cox proportional hazards regression model.^21^ Another nested cohort study noted that hysterectomy did not increase the risk of death from CVD; however, that study reported only mortality as the end point and did not specify the status of oophorectomy in women with hysterectomy.^22^ Population-based studies in the Olmsted County, Minnesota, region^23^ and Australia^24^ noted that hysterectomy without oophorectomy performed in women younger than 35 years was associated with an increased risk of coronary heart disease^23^ and cardiovascular mortality.^24^ In the Olmsted County cohort study, the risk of stroke was not significantly different between the hysterectomy and nonhysterectomy groups.^23^ However, there was no consideration of the use of MHT that could affect cardiovascular outcomes in that study. On the contrary, a nationwide, population-based study from Taiwan reported that women who underwent hysterectomy with ovarian conservation when younger than 45 years had an increased risk of stroke, while there was no significant difference of CVD risk after age 45 years.^25^ That result was analyzed after excluding women who had undergone menopause or those receiving MHT. As with the above study, we noted that, in women younger than 50 years, hysterectomy was independently associated with an increased risk of CVD, especially stroke. Our study adjusted for the use of MHT before and after inclusion. The result was consistent even after excluding patients who received oophorectomy. These findings suggest that the uterus may have a cardiovascular protective effect in women, independent of female sex hormones.

The pathobiologic mechanism by which hysterectomy increases the risk of CVD in women before the age of menopause remains uncertain. It has been previously proposed that one of the possible mechanisms is disruption of ovarian blood flow from ovarian ligaments during hysterectomy, which may result in premature ovarian failure.^21^ Decreased ovarian blood flow and low ovarian sex steroid levels have been noted after hysterectomy.^26,27,28^ However, there is controversy about whether ovarian function changes after hysterectomy; some studies have shown unchanged ovarian function after hysterectomy and salpingectomy.^29,30^

Another possible mechanism is that the loss of menstruation after hysterectomy may result in a hemorheologic deleterious effect. After menopause, an increase in hematocrit levels occurs. Elevated hematocrit levels are associated with increased blood viscosity, leading to endothelial injury, rupture of plaques by increasing shear stress on the vessel wall, and thrombus formation by red blood cell aggregation,^8,9,10^ thereby increasing the risk of adverse cardiovascular events. Several epidemiologic studies reported that a high hematocrit level might be related to greater risks for MI, stroke, and cardiovascular mortality in the general population.^31,32,33^ In addition, an increased hematocrit level can lead to excessive iron and ferritin levels, which is associated with increased hydroxyl radical production and progression of atherosclerosis.^11^ Furthermore, frequent phlebotomy via voluntary blood donation in healthy men can reduce their heme iron and ferritin levels by 44% and lead to reduction in CVD risk.^34,35,36^ Before menopause, women have substantially lower hematocrit, iron, and ferritin levels than men. Serum ferritin levels increase in postmenopausal women and this upward curve almost coincides with the curve of increasing CVD incidence after menopause.^37,38^ These observational studies suggest that early hysterectomy may cause hematocrit and ferritin level increases and thereby increase the risk of early CVD.

Using data from the HIRA, we noted that women who underwent hysterectomy before the average age of menopause had a higher risk of CVD compared with women with an intact uterus. Since the data regarding hematocrit or serum ferritin levels in this cohort population were limited, it could not be verified whether hemorheologic changes after hysterectomy affect the CVD risk. Further studies are needed to clarify the association between hysterectomy and hematocrit, iron, and ferritin levels and its possible effect on cardiovascular outcomes. Furthermore, it is difficult to explain why the risk of stroke appears to be significantly increased, while the risk of coronary artery disease and MI are not significantly different. Further mechanistic studies are required to clarify the noted association.

### Limitations

This study has several limitations. First, this was a retrospective, observational study. Second, since the study population comprised a homogeneous group of women of Korean ethnicity, the findings may not be generalizable. Third, studies using administrative databases are prone to errors caused by inaccurate coding.^39^ To minimize this problem, we applied definitions that were already validated in previous studies using the Korean NHIS database.^40^ Fourth, the inability to determine the severity of the underlying disease or treatment status that can affect the primary outcome may bias the study results. In addition, there was no consideration for time-varying covariates, such as MHT duration or treatment for CVD risk factors after inclusion, which could act as a residual confounder. Fifth, although propensity score matching was performed by categorizing the age groups as 40 to 44 and 45 to 49 years to reduce the confounding variable according to the wide age range, stratification of age groups into tighter bands would have reduced the age-dependent confounding effect. Sixth, some factors, such as body mass index and family history, could not be corrected since the corresponding data were limited. Seventh, the association between increased blood viscosity or ferritin levels with early hysterectomy and their outcomes could not be evaluated because data regarding hematocrit or ferritin levels were omitted. Eighth, because data on female sex hormone levels were absent, it was difficult to note whether hysterectomy is associated with the risk of CVD independently of female sex hormones. However, the subgroup analysis that excluded women who underwent salpingo-oophorectomy showed results that are consistent with those of our main analysis. Further research is needed on the mechanisms for the effects of the uterus on women’s cardiovascular health.

## Conclusions

In this cohort study of Korean women, we noted that hysterectomy in women younger than 50 years was independently associated with an increased risk of stroke. A difference of the risk of MI or coronary artery revascularization was not detected. Although we found that widely performed hysterectomy with a broad indication for benign diseases at premenopausal ages slightly increases the risk of CVD, the incidence is not high, so a change in clinical practice may not be needed.

## Notes

Supplement 1. **eTable 1.** Case/Person-Years of Cardiovascular Disease According to Hysterectomy **eTable 2.** Subgroup Analysis for the Risk of Cardiovascular Outcomes According to Whether Adnexal Surgery Was Performed Simultaneously With Hysterectomy **eTable 3.** Characteristics of Participants According to Whether or not Hysterectomy Was Performed (Non-Hysterectomy Versus Laparoscopic Hysterectomy) (Sensitivity Test) **eTable 4.** Hazard Ratios for Risk of Cardiovascular Disease (Laparoscopic Hysterectomy vs Non-Hysterectomy) **eFigure.** Kaplan-Meier Curves of Individual Outcomes

Supplement 2.
Data Sharing Statement
