# Supplementary material for: Association of Early Hysterectomy With Risk of Cardiovascular Disease in Korean Women
Source: JAMA Netw Open. 2023 Jun 12;6(6):e2317145. doi: 10.1001/jamanetworkopen.2023.17145 (PMC10261994; doi:10.1001/jamanetworkopen.2023.17145)
Supplement: Supplement 1. — eTable 1. Case/Person-Years of Cardiovascular Disease According to Hysterectomy eTable 2. Subgroup Analysis for the Risk of Cardiovascular Outcomes According to Whether Adnexal Surgery Was Performed Simultaneously With Hysterectomy eTable 3. Characteristics of Participants According to Whether or not Hysterectomy Was Performed (Non-Hysterectomy Versus Laparoscopic Hysterectomy) (Sensitivity Test) eTable 4. Hazard Ratios for Risk of Cardiovascular Disease (Laparoscopic Hysterectomy vs Non-Hysterectomy) eFigure. Kaplan-Meier Curves of Individual Outcomes [file jamanetwopen-e2317145-s001.pdf]

Supplementary Online Content

Yuk JS, Kim BG, Lee BK, et al. Association of early hysterectomy with risk of cardiovascular disease in Korean women. *JAMA Netw Open*. 2023;6(6):e2317145. doi:10.1001/jamanetworkopen.2023.17145

eTable 1. Case/Person-Years of Cardiovascular Disease According to Hysterectomy

eTable 2. Subgroup Analysis for the Risk of Cardiovascular Outcomes According to Whether Adnexal Surgery Was Performed Simultaneously With Hysterectomy

eTable 3. Characteristics of Participants According to Whether or not Hysterectomy Was Performed (Non-Hysterectomy Versus Laparoscopic Hysterectomy) (Sensitivity Test)

eTable 4. Hazard Ratios for Risk of Cardiovascular Disease (Laparoscopic Hysterectomy vs. Non-Hysterectomy)

eFigure. Kaplan-Meier Curves of Individual Outcomes

This supplementary material has been provided by the authors to give readers additional information about their work.

© 2023 Yuk JS et al. *JAMA Network Open.*

eTable 1. Case/person-years of cardiovascular disease according to hysterectomy.

	Case/person-years (case/100,000 person-years)	
	Non-Hysterectomy	Hysterectomy
Total	422/438,575 (96)	507/440,616 (115)
Age at inclusion (years)		
40~44	166/211,702 (78)	198/207,985 (95)
45~49	256/226,873 (113)	309/232,631 (133)
SES		
Mid~high SES	396/424,370 (93)	479/427,254 (112)
Low SES	26/14,205 (183)	28/13,362 (210)
Region		
Urban area	209/241,839 (86)	259/247,866 (104)
Rural area	213/196,736 (108)	248/192,750 (129)
CCI		
0	289/335,319 (86)	352/337,896 (104)
1	72/62,861 (115)	88/60,407 (146)
≥2	61/40,395 (151)	67/42,313 (158)
Parity in cohort		
0	412/430,487 (96)	501/432,497 (116)
1	3/5,729 (52)	4/5,707 (70)
≥2	7/2,359 (297)	2/2,412 (83)
Hypertension before inclusion		
Absent	315/391,129 (81)	379/391,632 (97)
Present	107/47,445 (226)	128/48,984 (261)
DM before inclusion		
Absent	369/409,268 (90)	439/411,259 (107)
Present	53/29,307 (181)	68/29,358 (232)
Dyslipidemia before inclusion		
Absent	325/366,523 (89)	398/369,317 (108)
Present	97/72,052 (135)	109/71,299 (153)
Menopause before inclusion		
Absent	375/400,994 (94)	460/403,977 (114)
Present	47/37,581 (125)	47/36,639 (128)
MHT before inclusion		
Absent	412/434,177 (95)	499/435,830 (114)
Present	10/4,398 (227)	8/4,786 (167)
Adnexal surgery before inclusion		
Absent	417/431,330 (97)	498/433,374 (115)
Present	5/7,245 (69)	9/7,242 (124)
First MHT after inclusion		
Absent	406/406,822 (100)	465/381,739 (122)
Present	16/31,753 (50)	42/58,877 (71)
First antithrombotic agent after inclusion		
Absent	363/423,196 (86)	446/423,236 (105)
Present	59/15,379 (384)	61/17,380 (351)

Data are expressed as the case/person-years (case/ 100,000 person-years). DM, diabetes mellitus; CCI, Charlson comorbidity index; MHT, menopausal hormone therapy; SES, socioeconomic status.

© 2023 Yuk JS et al. *JAMA Network Open.*

eTable 2. Subgroup analysis for the risk of cardiovascular outcomes according to whether adnexal surgery was performed simultaneously with hysterectomy.

Cardiovascular outcomes	No.(%) of event		HR (95% CI)[a]
	Non-hysterectomy (n=55,539)	Hysterectomy (n=55,539)	
**Cardiovascular disease**			
Hysterectomy without adnexal surgery	422 / 55,539 (0.8)	406 / 44,593 (0.9)	1.24 (1.06-1.44)
Hysterectomy with adnexal surgery	422 / 55,539 (0.8)	101 / 10,946 (0.9)	1.32 (0.97-1.79)
**Myocardial infarction**			
Hysterectomy without adnexal surgery	26 / 55,539 (0)	22 / 44,593 (0)	1.03 (0.53-2.01)
Hysterectomy with adnexal surgery	26 / 55,539 (0)	3 / 10,946 (0)	1.52 (0.15-15.87)
**Coronary artery revascularization**			
Hysterectomy without adnexal surgery	110 / 55,539(0.2)	94 / 44,593 (0.2)	1.10 (0.77-1.57)
Hysterectomy with adnexal surgery	110 / 55,539 (0.2)	18 / 10,946 (0.2)	0.78 (0.36-1.66)
**Stroke**			
Hysterectomy without adnexal surgery	322 / 55,539 (0.6)	322 / 44,593 (0.7)	1.28 (1.08-1.52)
Hysterectomy with adnexal surgery	322 / 55,539 (0.6)	84 / 10,946 (0.8)	1.43 (1.01-2.01)

Abbreviations: CI, confidence interval; HR, hazard ratio.

Adnexal surgery means the surgery of ovary or fallopian tubes.

[a] Adjusted for age, socioeconomic status, region, Charlson comorbidity index, parity, hypertension, diabetes, dyslipidemia, menopause before inclusion, menopausal hormone therapy before inclusion, adnexal surgery before inclusion and menopausal hormone therapy after inclusion.

© 2023 Yuk JS et al. *JAMA Network Open.*

eTable 3. Characteristics of participants according to whether or not hysterectomy was performed (non-hysterectomy versus laparoscopic hysterectomy) (Sensitivity test)

Characteristics	Participants, No. (%)			P-value
	Total (n=72,158)	Non-hysterectomy (n=55,539)	Laparoscopic hysterectomy (n=16,619)	
Age at inclusion, y				.05
40–44	34,725 (48.1)	26,615 (47.9)	8,110 (48.8)	
45–49	37,433 (51.9)	28,924 (52.1)	8,509 (51.2)	
Year at inclusion				<.001
2011	19,364 (26.8)	13,559 (24.4)	5,805 (34.9)	
2012	19,930 (27.6)	13,291 (23.9)	6,639 (39.9)	
2013	18,413 (25.5)	14,819 (26.7)	3,594 (21.6)	
2014	14,451 (20)	13,870 (25)	581 (3.5)	
Socioeconomic status				<.001
Receipt of medical aid as medical insurance	2,714 (3.8)	1,758 (3.2)	956 (5.8)	
Region				<.001
Urban area	40,807 (56.6)	30,995 (55.8)	9,812 (59)	
Rural area	31,351 (43.4)	24,544 (44.2)	6,807 (41)	
CCI				.20
0	55,133 (76.4)	42,501 (76.5)	12,632 (76)	
1	10,394 (14.4)	7,960 (14.3)	2,434 (14.6)	
≥2	6,631 (9.2)	5,078 (9.1)	1,553 (9.3)	
Hypertension	7,773 (10.8)	6,065 (10.9)	1,708 (10.3)	.02
Diabetes	4,888 (6.8)	3,756 (6.8)	1,132 (6.8)	.83
Dyslipidemia	12,005 (16.6)	9,268 (16.7)	2,737 (16.5)	.44
Menopause before inclusion	6,176 (8.6)	4,672 (8.4)	1,504 (9.0)	.001
MHT before inclusion	748 (1)	569 (1)	179 (1.1)	.56
Adnexal surgery before inclusion	1,213 (1.7)	952 (1.7)	261 (1.6)	.21

Abbreviations: CCI, Charlson comorbidity index; MHT, menopausal hormone therapy

© 2023 Yuk JS et al. *JAMA Network Open.*

eTable 4. Hazard ratios for risk of cardiovascular disease (laparoscopic hysterectomy vs. non-hysterectomy).

	HR (95% CI) *	P-value
Cardiovascular disease	1.17 (0.93-1.48)	0.191
Myocardial infarction	0.65 (0.21-2.04)	0.462
Coronary artery revascularization	0.87 (0.53-1.44)	0.592
Stroke	1.32 (1.01-1.72)	0.042

*Adjusted for age, socioeconomic status, region, Charlson comorbidity index, parity, hypertension, diabetes, dyslipidemia, menopause before inclusion, menopausal hormone therapy before inclusion, adnexal surgery before inclusion and menopausal hormone therapy after inclusion. CI, confidence interval; HR, hazard ratio

© 2023 Yuk JS et al. *JAMA Network Open.*

eFigure. Kaplan-Meier curves of individual outcomes.

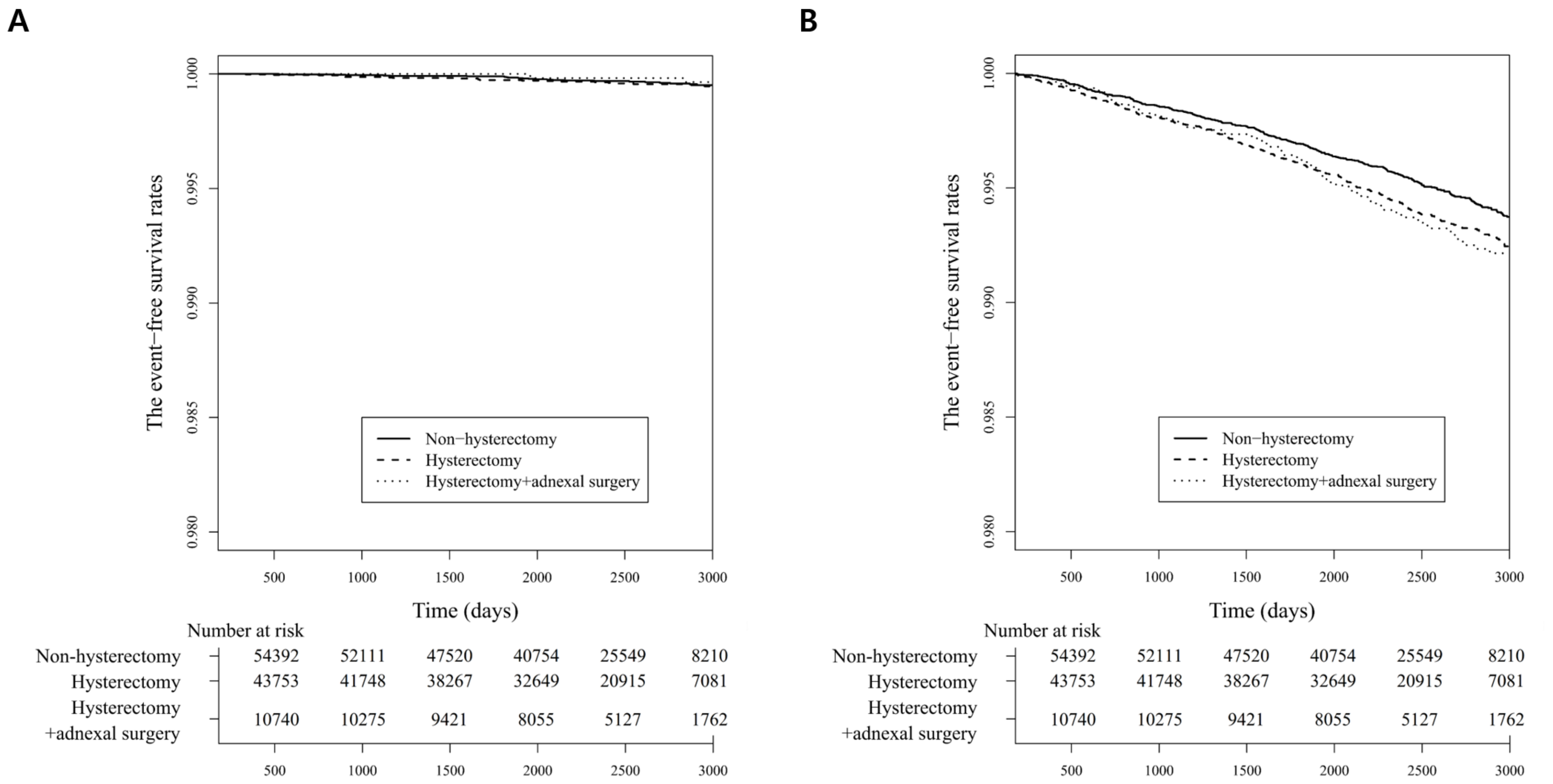

Kaplan-Meier curves for myocardial infarction (A) and stroke (B) according to hysterectomy status.

© 2023 Yuk JS et al. *JAMA Network Open.*
