# Supplementary material for: Association of Early Hysterectomy With Risk of Cardiovascular Disease in Korean Women
Source: JAMA Netw Open. 2023 Jun 12;6(6):e2317145. doi: 10.1001/jamanetworkopen.2023.17145 (PMC10261994; doi:10.1001/jamanetworkopen.2023.17145)
Supplement: Supplement 2. — Data Sharing Statement [file jamanetwopen-e2317145-s002.pdf]

Data Sharing Statement

Yuk. Association of Early Hysterectomy With Risk of Cardiovascular Disease in Korean Women. *JAMA Netw Open*. Published June 12, 2023.
doi:10.1001/jamanetworkopen.2023.17145

Data
Data available: No
